# On or Off: Life-Changing Decisions Made by *Vibrio cholerae* Under Stress

**DOI:** 10.1097/IM9.0000000000000037

**Published:** 2020-10-14

**Authors:** Yitian Zhou, Zachariah L. Lee, Jun Zhu

**Affiliations:** Department of Microbiology, Perelman School of Medicine, University of Pennsylvania, Philadelphia, USA.

**Keywords:** posttranscriptional modification, stress responses, transcriptional regulation, *Vibrio cholerae*, virulence

## Abstract

*Vibrio cholerae*, the causative agent of the infectious disease, cholera, is commonly found in brackish waters and infects human hosts via the fecal-oral route. *V. cholerae* is a master of stress resistance as *V. cholerae's* dynamic lifestyle across different physical environments constantly exposes it to diverse stressful circumstances. Specifically, *V. cholerae* has dedicated genetic regulatory networks to sense different environmental cues and respond to these signals. With frequent outbreaks costing a tremendous amount of lives and increased global water temperatures providing more suitable aquatic habitats for *V. cholerae*, cholera pandemics remain a probable catastrophic threat to humanity. Understanding how *V. cholerae* copes with different environmental stresses broadens our repertoire of measures against infectious diseases and expands our general knowledge of prokaryotic stress responses. In this review, we summarize the regulatory mechanisms of how *V. cholerae* fights against stresses in vivo and in vitro.

## Introduction

*Vibrio cholerae* is a facultative anaerobic, motile, gram-negative bacterium. It acquires virulence through toxin co-regulated pili (TCP) by incorporating the cholera toxin (CT) gene encoded by the filamentous phage, CTXϕ. The resulting lysogenic *V. cholerae* that is now capable of producing CT becomes toxigenic.^1^ This amenable acquisition of virulence through horizontal gene transfer has resulted in a large pool of potentially virulent *V. cholerae*, with two toxigenic serotypes, O1 and O139, being responsible for the majority of cholera cases worldwide.^2^*V. cholerae* serotypes are differentiated by their highly variable O antigens, which are vital for bacteriophage and mammalian immune system recognition and evasion.^3^ O139 is an O1 derivative with multi-gene substitutions in the O antigen-coding region.^4^ O1 has two distinct biotypes, the classical and El tor, that differ in phenotypes.^5^ The classical strain was the causative agent for cholera pandemics up until the early 20th century, while El tor has been the dominant strain in the past 30 years. The world is currently in its 7th cholera pandemic, with the disease being especially prevalent in resource-challenged regions, primarily in Africa and South and Southeast Asia, where access to clean drinking water is limited. Recent outbreaks in Yemen and Haiti, where infrastructure essential to the collection, treatment, and discharge of sewage was destroyed due to wars or earthquakes, have also demonstrated the effects of such calamitous situations in manifesting cholera outbreaks in otherwise non-historically endemic regions.

When a human host consumes food or water contaminated by the virulent *V. cholerae*, the pathogen penetrates the mucosal layer that covers the villi and then colonizes the intestinal tract. Upon colonization, *V. cholerae* produces virulence factors, TCP and CT. In addition to serving as the recognition surface structure for CTXϕ, TCP also facilitates the aggregation of bacteria and the tethering of cells to the host intestinal mucus layer as microcolonies. These multi-cell structures help combat the shearing forces of peristalsis in the small intestine and improve colonization.^6^ CT is a secreted AB_5_ multi-unit toxin. The pentameric subunit B binds to the enterocytes, which leads to endocytosis of the toxin, upon which subunit A becomes active and catalyzes the ADP-ribosylation of the host G protein. This in turn retains the G protein in a constant guanosine triphosphate (GTP)-bound form, causing continual adenylyl cyclase activity and cyclic adenosine monophosphate (cAMP) production in the host. The elevated cAMP levels inhibit sodium chloride absorption, promote chloride and bicarbonate secretion, and activate the cystic fibrosis transmembrane conductance regulator.^7^ These events cause an extensive efflux of electrolytes and fluid from infected enterocytes, leading to diarrhea, which allows *V. cholerae* to exit the host and return to an aquatic environment. For the patient, the profuse diarrhea causes potentially deadly dehydration and a loss of electrolytes leading to hypotonic shock. As such, the most direct and potent cure is rehydration by replacement of lost water and electrolytes.^2^ Even individuals with acquired immunity through either vaccination or past infection do not obtain life-long complete immunity.^8^ Effective containment of an outbreak still relies on waste management, chlorination of water, and frequent hand washing.^9^

## Navigating the aquatic environment: watch and roll with the punches

Many bodies of brackish waters with a relatively warm temperature above 15°C are suitable *V. cholerae* habitats.^10,11^ Some *V. cholerae* may never enter a mammalian host in their entire life. However, all *V. cholerae*, while living in an aquatic environment, need constant attendance to the numerous stresses such as nutrient scarcity, fluctuations of temperature and salinity, antibiotics secreted by other aquatic bacteria, and predatory behaviors such as protozoan grazing and vibriophage infections.^12-15^

One of the strategies used by *V. cholerae* and many other bacterial pathogens is to employ two-component systems (TCSs) to perceive and respond to the environmental stresses. In fact, TCSs are signaling architectures utilized by organisms across all three kingdoms to modulate biological activities according to the perceived environment.^16^ Upon encountering a positive external stimulus, the first component, the sensor histidine kinase (HK), autophosphorylates on a histidine residue and subsequently phosphorylates the second component, the cytosolic response regulator (RR), that responds via transcriptional regulation. *V. cholerae* encodes 52 RRs,^17^ many of which are involved in lifestyle switches in response to stressors. The PhoR/B TCS monitors periplasmic orthophosphate levels to facilitate dissemination at low phosphate concentrations.^18^ The CpxA/R TCS, triggered by envelope stress signaled by low extracellular iron or high extracellular chloride or copper,^19–21^ represses virulence and promotes efflux pump production to disgorge the stressor compounds.^20–22^ The HK ChiS activates the chitin utilization pathway central to environmental *V. cholerae* metabolism. Aquatic *V. cholerae* is often found to be associated with exoskeletons of crustaceans and soft-shelled turtles.^23–27^ These exoskeletons are rich in chitin, which are insoluble *N*-Acetylglucosamine (GlcNAc, or NAG) polymers. Via secreted chitinases, *V. cholerae* breaks down the chitin polymers and use the oligomers, detected by the HK ChiS, as their sole source of carbon and nitrogen.^28–30^

Multikinase networks (MKNs), pathways in which signals sensed by multiple HKs feed into a single regulation cascade,^31^ are exemplified by *V. cholerae's* quorum sensing (QS) regulation. At low cell densities, the absence of QS signal ligands, intra-genus QS signal CAI-1 for HK CqsS and inter-species QS signal AI-2 for HK LuxQ, activates both apo-HK to phosphorylate the phosphotransfer shuttle protein, LuxU, which relays the phosphorylation to the RR LuxO. Phosphorylated LuxO restricts the levels of the QS activator HapR by activating the transcription of the Qrr sRNAs that destabilize the *hapR* mRNA. The VarS/A TCS is a third input for LuxO. At low cell densities, both the HK and RR are inactive, resulting in the lack of transcription of inhibitory sRNAs that act on CsrA, a LuxO activity enhancer, therefore resulting in low levels of HapR.^32^ Certain molecules accumulated in the stationary phase activate the VarS/A TCS, which is also necessary for virulence.^33^ Although the VarS/A TCS acts independently of LuxU, LuxU also integrates the phosphorylation signals from two additional HKs: the cytosolic hybrid HK VpsS responding to nitric oxide, and the membrane-bound HK CqsR responding to the membrane phospholipid metabolite ethanolamine.^34–36^

At high cell densities, any of the four QS HKs feeding into LuxU can act as the sole activator for the QS circuit.^37^ As a result, multiple parallel sensory inputs indicating high cell density result in high levels of HapR, which repress both biofilm production and virulence. ^38,39^ Although not a stress response per se, the robust QS response at high cell densities ensures the activation of collective behaviors that benefit the population, such as exogenous DNA uptake that can diversify the gene pool and HA/protease activities that facilitate penetration through the mucus layer, detachment from host cells, and nutrient acquisition outside of the mammalian host.^40–43^

The wide fluctuations of temperature and salinity in the aquatic environment challenge *V. cholerae* homeostasis. In defense, *V. cholerae* often resorts to forming a biofilm under such stresses.^44–46^ Much like biofilms formed by other bacteria, *V. cholerae* biofilm is composed of cell aggregates and an extracellular matrix primarily consisting of polysaccharides (*Vibrio* polysaccharide, VPS), phospholipids, proteins, and extracellular DNA.^47^ Biofilm VPS production enables a wrinkled morphology, expanding the multi-cell community beyond a two-dimensional surface into a 3D structure that makes nutrients and signaling molecules more available to individual cells in the biofilm.^48–50^ Further improving their fitness, *V. cholerae* in an environmental biofilm employs a Type VI secretion system (T6SS) to kill neighboring cells and acquire their released DNA.^51^

Although biofilm formation confers resistance to many stressors, it is a transformative shift in which the individual cells are not maximizing their own fitness but rather that of the greater community. If the decision to form or stay in a biofilm is not made after a comprehensive assessment of the current situation, and other preferable defense mechanisms exist, it will be a costly, if not deadly choice for the entire community. For example, *V. cholerae* failing to exit a biofilm after entering the host small intestine have a colonization disadvantage compared to those that are planktonic.^52^ Therefore, biofilm formation is generally repressed by the histone-like nucleoid structuring proteins,^53^ which repress the expression of the *vps* operons as well as VpsT, one of the two biofilm master activators. In addition, a network of regulators, including the other biofilm activator VpsR,^54^ virulence activator AphA,^55^ and QS activator HapR,^52,56^ strictly modulate biofilm formation. The behavior of these proteins is influenced by signaling molecules, specifically positive signals (p)ppGpp and c-di-GMP,^57,58^ and negative signal cAMP.^59^ The cellular levels of these small molecules are influenced by environmental inputs, such as host contact, redox potential, and carbon availability. Therefore, these environmental signals serve as inputs for the activation or termination of the biofilm lifestyle.

In addition to forming biofilms on biotic and abiotic detritus, environmental *V. cholerae* under stress may also enter a metabolically quiescent state that is “viable but non-culturable”.^60,61^ This dormant state might serve similar functions to spores formed by spore-forming bacteria that allow the bacteria to persist until they can resume normal metabolism.

*V. cholerae* is faced with many choices in life, each requiring a careful observation of the situation followed by an adequate response that initiates the recruitment of specific machineries to address the specific scenario. Running unnecessary or inappropriate programs is wasteful and harms the fitness of the bacteria, attributing to a disadvantage compared to better adapted competitors.

## Infecting the host: strategic offense and cautious defense

**Figure 1:**
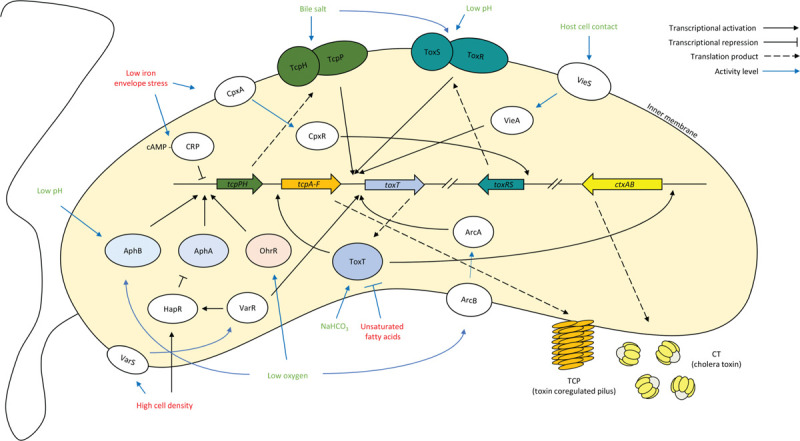
**Signaling network of *V. cholerae* virulence regulation**. A simplified schematic of the *V. cholerae* virulence regulatory network. Green signals have a net stimulating effect on virulence; red signals have a net repressive effect on virulence. Relationships indicated can be direct or via intermediate factors not shown.

When infecting a host, *V. cholerae* is faced with additional critical decisions. Not only do they need to survive the host environment, they also need to strategically turn virulence on and off for a successful colonization. To reach the primary colonization site, the small intestine, *V. cholerae* needs to endure the acid stress in the stomach and transition to a less oxygenated environment compared to aquatic reservoirs. Host signals reflecting this transition, such as changes in bile salt concentration,^62,63^ pH,^64,65^ unsaturated fatty acids,^66,67^ bicarbonate,^68^ iron concentration,^20,21^ and oxygen levels,^64^ collectively inform *V. cholerae* virulence regulation (Figure 1).

The membrane-bound transcription factor ToxR senses pH and bile salts that change its interaction dynamics with its stabilizing protein ToxS.^65^ In an acidic pH environment or in the presence of bile salts, enhanced ToxR-ToxS interaction activates ToxR regulatory functions, facilitating an outer membrane porin composition for organic acid resistance while turning on the virulence activator gene *toxT*.^69–72^ Another membrane-bound virulence activator TcpP, upon exposure to the bile salt taurocholate, transitions from a monomeric state to an transcriptionally active dimeric form with an intermolecular disulfide bond.^73^ Similar to ToxS stabilizing ToxR, TcpH, encoded in the same operon as *tcpP*, provides protection against proteolysis for TcpP.^74,75^ Activated by host signals, ToxR and TcpP collectively bind to the promoter region of *toxT* to initiate the transcription of the cytosolic master virulence activator, ToxT.^76–78^ ToxT in turn enters a positive feedback loop where it promotes its own expression all the while activating the expression of *ctxAB* and *tcpA*, which encode the two major virulence factors, CT and TCP, respectively. Further upstream in the regulatory cascade are cytosolic regulators AphB and AphA, which activate the transcription of the *tcpPH* operon collaboratively.^79^

To activate *tcpPH* expression, AphB requires both low pH and low oxygen concentrations as stimulating inputs.^80^ AphB is transcriptionally inactive at alkaline pH sensed by key residues in its ligand-binding pocket.^81^ At low pH, AphB becomes active and also activates the expression of *cadC*, which encodes an activator for the lysine decarboxylation machinery that consumes protons to increase the cellular pH for acid tolerance.^64^ Oxygen levels are sensed by AphB through the oxidation state of Cys235 in the C-terminal regulatory domain. Oxidation at Cys235 prevents AphB oligomerization, while a more anoxic environment leads to the reduction of this cysteine residue, facilitating AphB oligomerization necessary for *tcpPH* transcription.^80^ Therefore, AphB ensures that virulence is only turned on when the bacteria have survived the acid barrier in the stomach and reached the microaerobic environment of the small intestine. To activate *tcpPH* expression, AphB binds cooperatively with AphA at the *tcpPH* promoter.^79,82^ Since the expression of *aphA* is repressed by the QS regulator HapR,^83^ AphA serves as an indirect cell density sensor for virulence activation. At low cell densities, AphA levels are high, promoting virulence production; at high cell densities, AphA levels are reduced due to increased levels of HapR, therefore contributing to the inverse relationship between *V. cholerae* virulence and QS response.

Another regulator at the *tcpPH* promoter is cAMP-CRP,^84^ which inhibits the transcription of *tcpPH* when intracellular cAMP is abundant. Envelope stress, usually signaled by a lack of environmental iron or efflux components, alters carbon uptake and utilization,^20^ increasing levels of intracellular cAMP that enhances the cAMP-CRP interaction. The resulting complex binds to the *tcpPH* promoter as a repressor.^21^ Virulence repression facilitated by cAMP-CRP allows for the prioritization of external stresses such as envelope stress or a lack of a preferred carbon source over virulence induction.

Decreased oxygen level is an important signal to *V. cholerae* not only because it is an activating signal for virulence through AphB, but also because it signifies the need for a different collection of proteins. While *V. cholerae* can survive in a wide spectrum of oxygen levels, from fully aerobic to completely anaerobic, the differences on the proteomic level between *V. cholerae* under aerobiosis and anaerobiosis have revealed drastically different lifestyles under these respective conditions.^85^ Besides differences in proteins essential for the respective energy metabolism, aerobiosis is associated with more carbohydrate transporters while anaerobiosis is associated with more stress response proteins and fewer motility proteins such as the flagellin B subunit. Aside from changed amounts and classes of proteins, there are also spatial rearrangements of existing proteins in response to oxygen levels. For example, some chemotaxis-related proteins localize to polar and lateral membrane regions in microaerobiosis.^86^ Some of these oxic-to-anoxic transitions are mediated by global regulators such as the ArcB/A TCS,^87,88^ which regulates the transitions between the utilization of different electron transport strategies in respect to the redox environment.^89–91^ Upon sensing a more reduced inner membrane quinone pool, ArcB activates ArcA to regulate the expression of genes in the ArcA regulon,^90,91^ mostly repressing genes in the TCA cycle to minimize the generation of NADH, promoting glycolysis to push metabolism toward fermentation.^88^ Although these Arc TCS observations have not been made specifically in *V. cholerae*, the high homology between the *V. cholerae* and *Escherichia coli* Arc proteins suggests possible similar oxygen sensing mechanisms. A study by Sengupta and colleagues has suggested that *V. cholerae* ArcA activates the expression of virulence master activator *toxT* independently of ToxR and TcpP in the virulence regulatory network.^92^ The ArcA-dependent *toxT* expression is seen in both aerobiosis and anaerobiosis but is more pronounced in the latter,^92^ further corroborating microaerobiosis as a stimulating signal for *V. cholerae* virulence.

Host cell contact also serves as a positive signal for virulence. The *vieSAB* operon, repressed by the global repressor, the histone-like nucleoid structuring protein, and the QS regulator HapR,^93^ encodes the HK VieS, the RR VieA, and a third component, VieB.^94,95^ The VieS/A HK-RR pair, when activated by contact with intestinal epithelial cells,^96^ positively regulates the expression of the *vieSAB* operon and *toxT*. The RR VieA, in addition to its RR phosphorylation receiver domain and DNA-binding domain, has an EAL domain that hydrolyzes c-di-GMP when cells are adhered to the host epithelium. This hydrolyzation in turn induces *toxT* expression.^58,96^ Therefore, phosphorylated VieA relays the host signal to coordinate two negatively associated cellular processes: enhancing pathogenesis by *toxT* activation while interfering with biofilm maintenance by lowering c-di-GMP levels. The third component, VieB, contains the conserved aspartate residue for RR phosphorylation yet lacks a DNA binding domain. Instead, it contains a structural motif that facilitates protein interactions, allowing it to bind to VieS and inhibit its autophosphorylation.^97^ Therefore, at high transcriptional levels of the *vieSAB* operon, VieB accumulates, forming a negative feedback loop that terminates further stimulation of virulence from this avenue.

Cellular processes that impact virulence, either negatively or positively, are extensively studied. Besides QS, motility is also negatively associated with virulence.^98^ NQR, the sodium ion-translocating NADH: quinone oxidoreductase, oxidizes NADH to generate a sodium motive force that drives flagellar rotation and the exchange of cations critical for motility and the maintenance of the transmembrane voltage.^99^ A *nqr* mutant lacking this membrane potential shows diminished motility and biofilm formation but an increased production of virulence factors such as TCP. Conversely, processes that positively correlate with virulence include chemotaxis, lipopolysaccharide biosynthesis, cyclic dinucleotides modulation by CD-NTases within the *Vibrio* pathogenicity island,^100^ and the biotin and purine synthesis pathways.^101^ However, for some of these positively associated processes, it is still unclear whether their correlation with virulence indicates causality in either direction, i.e., if these processes activate virulence, or vice versa.

The proteolysis of the virulence master activator, ToxT, marks the termination of *V. cholerae* virulence.^102^ Before exiting the host as *V. cholerae* reaches the lower intestines, the extracellular environment shifts from virulence-inducing to virulence-repressing due to increased pH and temperature, thereby breaking the ToxT-autoregulatory loop via degradation of ToxT.

By monitoring the extracellular pH, oxygen tension, and host signals such as bile salts and cell contact, *V. cholerae* strategically turns on virulence only when the external signals indicate its presence at the primary colonization site.

## Response to oxidative stress: borrow the enemy's arrows to survive the enemy's attack

*V. cholerae* encounters unique challenges in the aquatic environment and in the host, but some stressors are present in both. Among these ubiquitous stressors are reactive oxygen species (ROS), oxygen containing molecules that are highly reactive due to their unpaired electron. In the environment, exogenous ROS sources include other microbes co-inhabiting a niche^103–105^ or completely non-biological processes such as photochemical reactions.^106,107^ In a host, ROS are produced by neutrophils, macrophages, and epithelial cells that use NADPH oxidases to reduce oxygen to superoxide anions and hydrogen peroxide.^104^ ROS can be non-radicals, such as hydrogen peroxide and superoxide, or more reactive and damaging radicals, such as the hydroxyl radical, superoxide anion, and singlet oxygen that can be generated by non-radical species in the presence of transition metals such as iron and copper through Fenton chemistry. To avoid the Fenton reaction, *V. cholerae* iron uptake is tightly regulated to ensure sufficient iron for normal functions of iron-requiring proteins while avoiding reactive species damage.^108^ Less prevalent but equally damaging are other classes of reactive molecules that damage cellular targets in manners similar to ROS. These species include reactive nitrogen species generated from nitrogen metabolism; reactive electrophile species generated from quinones and aldehydes; and hypochloric acid generated by host myeloperoxidase in the presence of H_2_O_2_ and Cl^–^.^109^

Due to their reactive nature, ROS cause oxidative damage to lipids, nucleic acids, and proteins. Upon peroxidation, lipids, which are fundamental to cell membranes, degrade into cytotoxic aldehydes and hydrocarbons.^110^ When in contact with nucleic acids, ROS can act on the sugar backbone, leading to single-strand breakage, or on the nucleobases, resulting in base degradation and the generation of free radicals that lead to DNA-protein cross-links.^111–113^ Some of these damages can be amended by DNA repair enzymes such as those recruited in the SOS repair response,^114,115^ but the low fidelity of SOS polymerases such as DinB can cause mutations with major consequences.^116^ In addition to the unintended cross-linking to DNA, proteins upon oxidative modification on amenable residues are subject to fragmentation, altered electrical charges, and conformational changes.^117,118^ Thus, in contrast to other stressors like nutrient scarcity and antibiotics, which only target specific metabolic processes and only act on actively metabolizing cells, reactive species attack both the blueprint and the building blocks of life, regardless of the metabolic state of the cells. This poses tremendous challenges for all living organisms. Despite self-harming consequences such as cancer,^119^ host cells use ROS as an effective weapon to eliminate pathogens.

**Figure 2:**
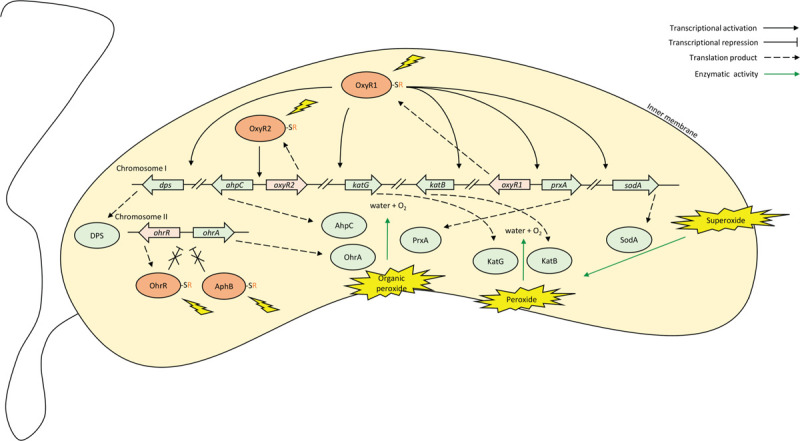
**Thiol-based regulation of *V. cholerae* ROS resistance**. A simplified schematic of the *V. cholerae* ROS regulatory network when challenged by ROS. Relationships indicated can be direct or via intermediate factors not shown. ROS: Reactive oxygen species.

To cope with this common stress, *V. cholerae* encodes many mitigating enzymes targeting different ROS (Figure 2). Superoxide dismutases such as the manganese-binding SodA convert superoxide into hydrogen peroxide and oxygen.^120^ Catalases such as KatB and KatG detoxify peroxides into water and oxygen.^121^ Peroxiredoxins such as PrxA and AphC target organic (alkyl) hydroperoxides.^122^ DNA-binding proteins from starve cells (DPS) physically bind to DNA to prevent ROS damage.^120^ The virulence regulator ToxR, activated by the host environment, promotes a proper intracellular manganese level for ROS resistance.^123^*V. cholerae* even resorts to elevating mutation frequencies to diversify and enrich ROS resistance enhancing phenotypes, such as increased catalase and VPS production.^124^

Although protein oxidation is generally undesirable as it often leads to misfolding or aggregation followed by removal and degradation, bacteria utilize oxidizable cysteine residues for protein functions.^125^ Cysteine has a highly nucleophilic thiol side chain that tends to donate electrons, especially to other sulfhydryl groups, to form a disulfide bond. Making up only 1.3% of all reported proteins in the UniProt database, cysteine is a low occurrence residue commonly reserved only for its irreplaceable functions. For example, all *V. cholerae* c-type cytochromes rely on correct disulfide bond formation between their cysteine residues for maturation and heme-interaction.^126^ In the case of heme nitric oxide/oxygen-binding (H-NOX) proteins, oxidation induces a conformational change through heme dissociation or disulfide bond formation at a zinc-binding motif encompassing four cysteine residues.^127^ The resulting H-NOX binds to the HK in the HnoK/B TCS, shutting down its kinase activity.^128^ The RR HnoB contains an EAL domain that hydrolyzes c-di-GMP when phosphorylated, decreasing the positive signal for biofilm formation.^129^ Therefore, the presence of oxidants causes H-NOX to inhibit the HnoK/B TCS, enhancing biofilm development.

Furthermore, reversible oxidation at cysteine residues is utilized to regulate redox stress response genes on a transcriptional level.^130–132^*V. cholerae* utilizes thiol-based transcription switches to adapt to different redox environments and their respective oxidative stress. As described above, the AphB microaerobiosis induction of virulence is contingent on reduced Cys235.^80^ In fact, the non-redox sensing *aphB* mutant strain-with Cys235 mutated to a serine-is more susceptible to ROS.^133^ AphB works closely with OhrR, another thiol-based transcription regulator that responds to redox changes sensed by its Cys23 and Cys128, in activating the transcription of the organic hydroperoxidase OhrA.^133^ Oxidized OhrR falls off of the promoter of *ohrA* faster than oxidized AphB, derepressing the ROS resistance gene sequentially in regard to the amount of ROS present. Therefore, *ohrA* is transcribed as demanded by the severity of the oxidative stress. Furthermore, the AphB OhrR duo exhibit the same differential kinetics when regulating the expression of the virulence regulator *tcpP*. Similar to Cys235 on AphB, Cys23 and Cys128 on OhrR in their reduced forms facilitate OhrR binding to the *tcpP* promoter, activating the transcription of the virulence activator. With multiple redox-sensing regulators, one responding more rapidly than the other, *V. cholerae* ensures a prudent initiation of virulence in a new redox environment.^134^

Besides OhrA, many other major ROS resistance enzymes are specifically upregulated upon ROS exposure, which is detected by the ROS-sensing regulators OxyR1 and OxyR2.^120,122^ Upon oxidation, sulfenation at the conserved cysteine residue Cys199 on the *E. coli* OxyR is critical for activating its regulatory functions.^135,136^ Two homologs of the *E. coli* OxyR, OxyR1 and OxyR2, both with the conserved redox-sensing cysteine residues, exist in the *V. cholerae* genome. OxyR1 responds to hydrogen peroxide and activates the expression of *prxA*, *katB*, *katG*, *sodA*, and *dps*, all of which contribute to ROS resistance.^120^ OxyR2 responds to environmental oxidative stress and activates the transcription of itself and the divergently transcribed *ahpC*, both of which promote *V. cholerae's* transition from the oxygen-limiting gut to oxygen-rich aquatic environments.^122^ Both OxyR1 and OxyR2 activations rely on the oxidation of the conserved cysteine residues equivalent to the *E. coli* OxyR Cys199. OxyR's regulatory function becomes inactive when the Cys199 thiol oxidation is reduced by enzymes in its regulon, forming an autoregulatory negative feedback loop. Through the different regulation dynamics of OxyR1 and OxyR2 responding to different oxidants, *V. cholerae* expresses ROS resistance enzymes adaptively and shut down the circuit when the oxidative stress is ameliorated. Figure 2 summarizes the complex regulatory network when *V. cholerae* is under ROS attack.

## Concluding remarks

*V. cholerae* stress responses exemplify the diverse strategies for bacterial survival across different physical environments. *V. cholerae* situates itself by perceiving environmental cues that directly describe the circumstance, such as oligosaccharide and phosphate levels, or resort to signals that stably correlate with the condition, such as an elevated bile salt level as a sign of arrival at a colonization site and an abundance of autoinducers as an indication of high cell density. The perception of these signals by cellular sensors feeds into programmed cellular circuits that yield a response tailored to maximize fitness in the environment reflected by those signals. The result is a streamlined process of stress exposure and stress response. Among these, the sensing of redox signals through oxidation states on cysteine thiols is especially critical as it modulates very common and dynamic processes. *V. cholerae* is not unique in employing this design in redox sensing. Therefore, the mechanism of *V. cholerae* thiol-based switches are instructive to a better understanding of bacterial redox sensing and the many events involved in bacterial ROS resistance. As a universal stress for all living organisms regardless of the metabolic state, ROS is a potent weapon against pathogens with its wide-range damage to multiple cell components. More knowledge on bacterial ROS resistance will add to the mechanistic underpinnings for the design and guidance of strategic anti-microbial interventions.

## Acknowledgments

The authors thank all past and present lab members for helpful discussions.
